# Nomograms to Predict Endocrinological Deficiency in Patients With Surgically Treated Craniopharyngioma

**DOI:** 10.3389/fonc.2022.840572

**Published:** 2022-05-19

**Authors:** Jie Wu, Xiao Wu, Le Yang, ShenHao Xie, Bin Tang, ZhiGao Tong, BoWen Wu, YouQing Yang, Han Ding, YouYuan Bao, Lin Zhou, Tao Hong

**Affiliations:** Department of Neurosurgery, The First Affiliated Hospital of Nanchang University, Nanchang, China

**Keywords:** craniopharyngioma, nomogram, endocrinological deficiency, hypothalamus–pituitary axis, pituitary stalk

## Abstract

**Objective:**

Postoperative hypopituitarism associated with increased risks of premature mobility and mortality is often encountered in craniopharyngioma patients. The aim of our study is to construct nomograms related to injury types of the hypothalamus–pituitary axis (HPA) to predict hypopituitarism 1 year after surgery.

**Methods:**

Craniopharyngioma patients undergoing initial endoscopic endonasal surgery between December 2012 and March 2021 in our center were retrospectively reviewed, and injury types of the HPA were categorized according to intraoperative endoscopic observation. Included patients were randomly divided into a training group and a validation group. Nomograms were established based on the results of multivariate logistic analysis. The predictive performance of the nomograms was evaluated in the training and validation groups.

**Results:**

A total of 183 patients with craniopharyngioma were enrolled, and seven injury types of the HPA were summarized. Relative to intact HPA, exclusive hypothalamus injury significantly increased the risk of anterior (OR, 194.174; 95% CI, 21.311–1769.253; p < 0.001) and posterior pituitary dysfunction (OR, 31.393; 95% CI, 6.319–155.964; p < 0.001) 1 year after surgery, while exclusively sacrificing stalk infiltrated by tumors did not significantly increase the risk of anterior (OR, 5.633; 95% CI, 0.753–42.133; p = 0.092) and posterior pituitary dysfunction (OR, 1.580; 95% CI, 0.257–9.707; p = 0.621) 1 year after surgery. In the training group, the AUCs of nomograms predicting anterior and posterior pituitary dysfunction 1 year after surgery were 0.921 and 0.885, respectively, compared with 0.921 and 0.880 in the validation group.

**Conclusions:**

Intact hypothalamus structure is critical in maintaining pituitary function. Moreover, our preliminary study suggests that the pituitary stalk infiltrated by craniopharyngioma could be sacrificed to achieve radical resection, without substantially rendering significantly worse endocrinological efficiency 1 year after surgery. The user-friendly nomograms can be used to predict hypopituitarism 1 year after surgery.

## Introduction

Endocrinological deficiency associated with increased risks of premature mobility and mortality (1–3) is often encountered after surgery secondary to the injury of the hypothalamus–pituitary axis (HPA) in patients with craniopharyngioma (CP) (4–6). Meanwhile, it is well understood that the HPA plays a pivotal role in maintaining pituitary function. However, when evaluating endocrinological outcomes following surgery or addressing the issue that whether the pituitary stalk infiltrated by tumors can be sacrificed to achieve radical resection, previous studies only analyzed the effects of either the injury of the pituitary stalk (7–11) or the injury of the hypothalamus (12, 13) or the injury of the pituitary (14) on pituitary function, not mentioning any information about whether or not the other part of the HPA was injured. Given that the combined injury of the HPA is often encountered in patients with surgically treated CP due to different topographical characteristics of tumors, it is necessary to elucidate the impacts of combined injuries of the HPA on pituitary function, which is very helpful to comprehensively evaluate the impacts of the manipulation of the HPA on pituitary function and further tailor individual surgical strategies. Besides, risk factors associated with postoperative hypopituitarism in patients with CP are not well understood, which are pivotal to making informed decisions prior to and during surgery, aiming to improve endocrinological outcomes. Further, to date, there have been no prediction models to predict endocrinological deficiency for CP patients during follow-up, which is helpful to tailor individual endocrinological follow-up plans for patients with a high rate of postoperative endocrinological deficiency.

To address these gaps in the literature, we categorized the injury of the HPA into seven types based on intraoperative endoscopic observation and constructed nomograms incorporating independent risk factors to predict endocrinological deficiency 1 year after surgery.

## Materials and Methods

### Patient Population

After obtaining the board approval of the local ethics committee, the medical files and imaging data of consecutive craniopharyngioma patients who underwent fully endoscopic endonasal approach (EEA) between December 2012 and March 2021 in our center were retrospectively reviewed. Inclusion criteria were listed as follows: (1) the diagnosis of craniopharyngioma was histologically confirmed; (2) medical records were complete, including demographic data, pre- and postoperative imaging data, preoperative assessment, tumor characteristics, intraoperative videos, surgical results and complications, postoperative management, follow-up, and endocrinological outcomes; and (3) endocrinological evaluation with a minimum 1-year follow-up was required. Patients undergoing reoperations were excluded because the possible initial manipulation of the HPA could affect the identification of the injury type of the HPA and the endocrinological outcomes. In addition, patients undergoing tumor recurrence or regrowth within 1 year of follow-up were excluded, because of possible effects on endocrinological outcomes. The manuscript conforms to the STROBE (Strengthening the Reporting of Observational Studies in Epidemiology) guidelines.

Tumor size estimated as the maximal tumor diameter, tumor location, tumor consistency, calcification, and hydrocephalus were determined by MR images and computed tomography (CT) scans before surgery. Regarding the extent of resection (EOR), gross total resection (GTR) was defined as no residual tumor evaluated by postoperative contrast-enhanced MR images acquired within 72 h after surgery. In contrast, cases in which ≥80% of the tumor was resected were deemed as subtotal resection (STR) (9).

**Figure 1:**
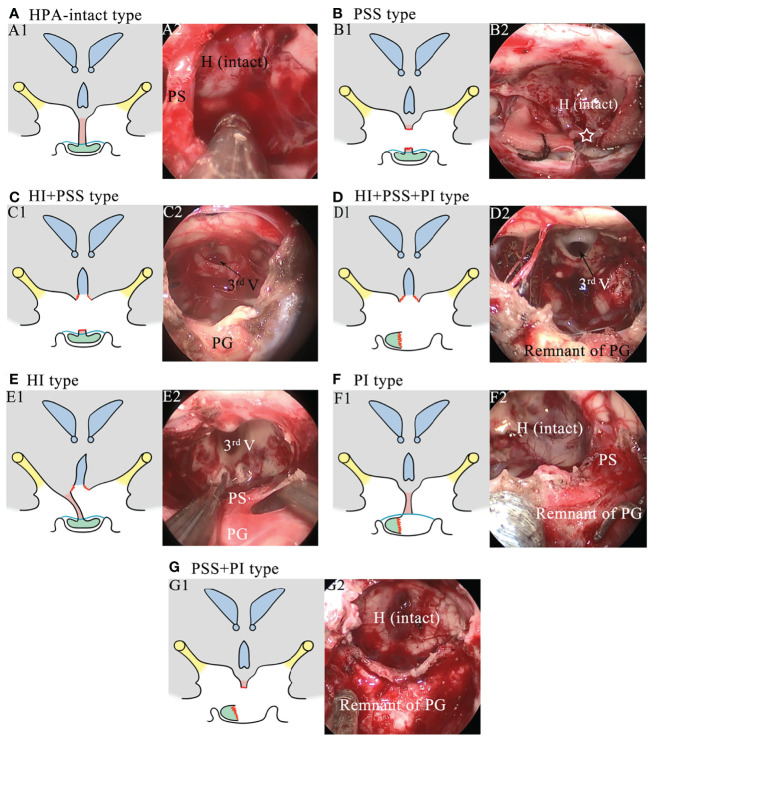
Schematics and intraoperative images showing the injury types of the hypothalamus–pituitary axis. **(A)** Schematic of the HPA-intact type (A1) and the corresponding intraoperative image (A2). The hypothalamus, the pituitary stalk, and the pituitary gland are intact. H, hypothalamus; PS, pituitary stalk. **(B)** Schematic of the PSS type (B1) and the corresponding intraoperative image (B2). The pituitary stalk is resected, while the hypothalamus and the pituitary gland are intact. The white star represents the stalk proximal stump. **(C)** Schematic of the HI+PSS type (C1) and the corresponding intraoperative image (C2). The third ventricle floor is open, and the pituitary stalk is resected, while the pituitary gland is intact. 3rd V, the third ventricle; PG, pituitary gland. **(D)** Schematic of the HI+PSS+PGI type (D1) and the corresponding intraoperative image (D2). The third ventricle floor is open, the pituitary stalk is resected, and the pituitary gland is injured, rendering a remnant of the pituitary gland. **(E)** Schematic of the HI type (E1) and the corresponding intraoperative image (E2). The third ventricle floor is open, while the pituitary stalk and the pituitary gland are preserved. **(F)** Schematic of the PGI type (F1) and the corresponding intraoperative image (F2). The pituitary gland is injured, while the hypothalamus and the pituitary stalk are preserved. **(G)** Schematic of the PSS+PGI type (G1) and the corresponding intraoperative image (G2). The hypothalamus is intact, while the pituitary stalk is resected and the pituitary gland is injured, rendering a remnant of the pituitary gland.

Regarding the injury of the HPA, seven injury categories, including intact HPA (HPA-intact type; **Figure 1A**), exclusive pituitary stalk sacrifice (PSS) type (**Figure 1B**), hypothalamic injury combined with pituitary stalk sacrifice (HI+PSS) type (**Figure 1C**), hypothalamic injury combined with pituitary stalk sacrifice and pituitary gland injury (HI+PSS+PGI) type (**Figure 1D**), exclusive hypothalamic injury (HI) type (**Figure 1E**), exclusive pituitary gland injury (PGI) type (**Figure 1F**), and pituitary stalk sacrifice combined with pituitary gland injury (PSS+PGI) type (**Figure 1G**), were defined based on intraoperative videos. For the purpose of our study, we defined HI as the lack of the integrity of the hypothalamus, including mild HI, unilateral HI, and bilateral HI, which we have previously described elsewhere (15). Given that the pituitary stalk partly anatomically preserved may show function, only patients subjected to complete transection of the PS were included into the PSS group. Patients with intrasellar CP or supradiaphragmatic CP extending into the intrasellar were deemed as having PGI because of inevitable intraoperative manipulation of the pituitary gland. The confirmation of injury types was conducted by two independent neurosurgeons who were blinded for clinical outcomes. If they failed to reach consensus, a third neurosurgeon was assigned to make the final decision.

### Endocrinological Evaluation

Presurgical and postsurgical endocrinological status was evaluated as described previously (16, 17). Partial hypopituitarism was defined as hormone deficiencies in one or two axes, and panhypopituitarism was defined as hormone deficiencies in three or more axes. Diabetes insipidus (DI) was evaluated preoperatively and postoperatively. Patients were diagnosed as having DI if they had polydipsia (excessive drinking; >3 l/day) and polyuria (excessive urination; >50 ml/kg body weight/24 h) combined with urine-specific gravity <1.005 and urine osmolality <300 mOsm/kg, as well as a positive response to desmopressin (15, 18). All the patients were recommended to receive imaging and clinical evaluation 3 months, 6 months, 12 months, and every 2 years subsequently after discharge. During follow-up, serum pituitary hormone levels were measured, and an endocrinologist analyzed the results. The protocol of hormone replacement was determined by the endocrinologist. Anterior pituitary dysfunction during follow-up was defined as replacement hormone required for at least one anterior pituitary hormone axis (14). For the purpose of our study, we focused on the endocrinological outcomes at 1 year after surgery and permanent DI representing posterior pituitary function was defined as at least 1 year of the need for desmopressin therapy after surgery (19).

### Surgical Procedure, Surgical Principle, and Adjuvant Treatment

All procedures were performed using an EEA. An image-guided neuronavigation system was employed for all procedures. All surgeries were performed by the chief surgeon (TH) and assisted by attending surgeons (BT, SHX, and LY). The surgical nuances of CP resection did not differ significantly from those described in the literature (20). After tumor resection, the closure was performed with a multilayered reconstruction, as described in previous literatures (21). Of note, the following surgical principles were strictly followed during CP resections. First, all procedures, especially in the process of tumor dissection, must be performed under direct visualization, avoiding pulling blindly. Second, carefully identifying and sharply combined with bluntly dissecting the cleavage plane between tumors and the HPA were warranted to achieve radical resection of CP and preserve the anatomical integrity of the structure of the HPA involved in the attachment to the greatest extent. When the stalk was infiltrated by tumors and extremely thin, the stalk would be sacrificed to achieve a radical resection. In addition, less aggressive resection was performed for patients with the extensive involvement of the hypothalamus or with adherence of a thin CP cyst capsule to critical vessels to avoid the higher risk of hypothalamic damage and fatal complications. Radiotherapy was routinely performed in CP patients undergoing STR 3 months after surgery.

### Statistical Analysis and Nomogram Construction

All patients were randomly divided into a training group and a validation group in a 7:3 ratio. Continuous variables were reported as mean (± standard deviation) or median with an interquartile range (IQR), which was determined by the Shapiro–Wilk test. Categorical data were reported as counts and proportions in each group. The baseline characteristics between the two groups were compared *via* the chi-square test (Fisher’s exact test where appropriate) for categorical variables and two-tailed Student (Mann–Whitney U-test where appropriate) for continuous variables. Interrater reliability for classifying the injury type of the HPA was performed using Cohen’s kappa, indicating a good level of agreement (κ = 0.781). Univariate and stepwise multivariate logistic regression was used to determine independent risk factors associated with the need for anterior pituitary hormone replacement 1 year after surgery and permanent DI in the training cohort. First, we performed univariate logistic regression analysis using all the clinical variables associated with endocrinological deficiency after CP surgery based on our subject matter knowledge and the established risk factors identified by previous literatures (7, 10, 14). Then, a stepwise multivariate logistic regression analysis was performed using variables with p < 0.1 in the univariate logistic regression analysis. A nomogram was drawn based on the results of the multivariate logistic regression analysis. To evaluate the discrimination efficiency and calibration power of the nomogram, the area under the receiver operating characteristic (ROC) curve (AUC) and calibration curve with 1,000 bootstrap samples were employed in the training cohort, respectively, and validated in the validation cohort. Regarding AUC, a value >0.8 was thought to have a good discrimination according to the grading guidelines (22). At last, for clinical use of the model, the total scores were calculated based on the nomogram for each patient. The procedures of conducting the nomograms followed the recommendations of “seven steps for development and an ABCD for validation” proposed by Steyerberg et al. (23).

SPSS (version 25.0; IBM Corp., Armonk, New York) was used to perform statistical analyses and plot ROC curves, while GraphPad Prism version 8 (GraphPad Software, La Jolla, CA) was used to plot histograms. R software (version 4.0.3; http://www.Rproject.org) was used to plot nomograms and calibration curves, with the “rms” package used. A two-sided level of p < 0.05 was considered statistically significant unless indicated otherwise.

## Results

### Clinical Characteristics

According to the inclusion and exclusion criteria, after excluding the patients undergoing reoperations, cases with perioperative mortality or loss to follow-up, and patients with endocrinological evaluation after surgery less than 1 year, 183 CP patients (104 men and 79 women) were enrolled into the final study cohort. The median follow-up duration of the entire cohort was 29.0 months (IQR 19–46 months). The included patients were randomly divided into the training (138 cases) and validation cohorts (45 cases).

Clinical data associated with risk of need for anterior pituitary hormone replacement 1 year after surgery and permanent DI are listed in **Table 1**. The baseline characteristics between these two cohorts were similar without significant difference. The number of permanent DI and needs for anterior pituitary hormone replacement 1 year after surgery was 71 (51.4%) and 94 (68.1%) in the training cohort, respectively, while 22 (48.9%) and 30 (66.7%) in the validation cohort, respectively.

**Table 1:** Participant characteristic.

Variable	Cohort, no. (%)		P value
	Training (n = 138)	Validation (n = 45)	
Age in years (median with IQR)	41.0 (24.8–53.0)	45.0 (23.0–54.0)	0.651
Sex			0.842
Male	79 (57.2)	25 (55.6)	
Female	59 (42.8)	20 (44.4)	
Age group			0.595
Child (<18 years)	20 (14.5)	8 (17.8)	
Adult (≥18 years)	118 (85.5)	37 (82.2)	
Preop BMI in kg/m^2^ (mean ± SD)	22.8 ± 3.7	23.1 ± 4.4	0.582
Preop hyperprolactinemia			0.636
Yes	68 (49.3)	24 (53.3)	
No	70 (50.7)	21 (46.7)	
Preop hydrocephalus			0.946
Yes	30 (21.7)	10 (22.2)	
No	108 (78.3)	35 (77.8)	
Preop DI			0.297
Yes	35 (25.4)	8 (17.8)	
No	103 (74.6)	37 (82.2)	
Preop pituitary function			0.897
Normal	47 (34.1)	14 (31.1)	
Partial hypopituitarism	67 (48.6)	22 (48.9)	
Panhypopituitarism	24 (17.4)	9 (20.0)	
Maximum diameter			0.573
≥3 cm	68 (49.3)	20 (44.4)	
<3 cm	70 (50.7)	25 (55.6)	
Tumor consistency			0.960
Solid	37 (26.8)	12 (26.7)	
Mixed	64 (46.4)	20 (44.4)	
Cystic	37 (26.8)	13 (28.9)	
Tumor location			0.071
Subdiaphragmatic	25 (18.1)	15 (33.3)	
Supradiaphragmatic	104 (75.4)	29 (64.4)	
Pure endoventricular	9 (6.5)	1 (2.2)	
Calcification			0.322
Yes	68 (49.3)	26 (57.8)	
No	7 0 (50.7)	19 (42.2)	
EOR			1.000
GTR	127 (92.0)	41 (91.1)	
STR	11 (8.0)	4 (8.9)	
Pathology			0.314
ACP	127 (92.0)	44 (97.8)	
PCP	11 (8.0)	1 (2.2)	
Postop hypernatremia			0.357
Yes	47 (34.1)	12 (26.7)	
No	91 (65.9)	33 (73.3)	
Postop radiotherapy			1.000
Yes	11(8.0)	4 (8.9)	
No	127 (92.0)	41 (91.1)	
Injury type			0.448
HPA-intact	26 (18.8)	5 (11.1)	
HI	25 (18.1)	8 (17.8)	
HI+PSS	36 (26.1)	10 (22.2)	
HI+PSS+PGI	12 (8.7)	6 (13.3)	
PSS	13 (9.4)	2 (4.4)	
PGI	17 (12.3)	8 (17.8)	
PGI+PSS	9 (6.5)	6 (13.3)	
Need for anterior pituitary hormone replacement 1 year after surgery			0.857
Yes	94 (68.1)	30 (66.7)	
No	44 (31.9)	15 (33.3)	
Permanent DI			0.765
Yes	71 (51.4)	22 (48.9)	
No	67 (48.6)	23 (51.1)	

IQR, interquartile range; BMI, body mass index; SD, standard deviation; DI, diabetes insipidus; GTR, gross total resection; STR, subtotal resection; ACP, adamantinomatous craniopharyngioma; PCP, papillary craniopharyngioma; HPA, hypothalamus–pituitary axis; HI, hypothalamic injury; HI+PSS, hypothalamic injury combined with pituitary stalk sacrifice; HI+PSS+PGI, hypothalamic injury combined with pituitary stalk sacrifice and pituitary gland injury; PSS, pituitary stalk sacrifice; PGI, pituitary gland injury; PGI+PSS, pituitary gland injury combined with pituitary stalk sacrifice.

### Risk Factors for Need for Anterior Pituitary Hormone Replacement 1 Year After Surgery

Variables including presurgical, surgical, and postsurgical factors were selected to determine candidate risk factors *via* univariate logistic regression analysis. In this step, preop pituitary function (p = 0.001), tumor location (p = 0.012), and injury type (p < 0.001) were identified as potential risk factors and further included into stepwise multivariate logistic regression analysis to determine independent risk factors associated with the need for anterior pituitary hormone replacement 1 year after surgery (**Table 2**). On multivariate analysis, with results reported as odds ratio (OR, 95% CI), preop pituitary function (for partial hypopituitarism vs. normal, 4.184 [1.225–14.292]; for panhypopituitarism vs. normal, 13.742 [1.345–140.417]) and injury type (for HI vs. HPA-intact, 194.174 [21.311–1769.253]; for HI+PSS vs. HPA-intact, 89.443 [13.642–586.416]; for HI+PSS+PGI vs. HPA-intact, 68.111 [4.975–932.565]; for PSS vs. HPA-intact, 5.633 [0.753–42.133]; for PGI vs. HPA-intact, 159.790 [11.974–2132.357]; for PSS+PGI vs. HPA-intact, 31.921 [3.320–306.873]) were determined as independent risk factors and further used to form a nomogram (**Table 3**). Intriguingly, the difference in the risk of need for anterior pituitary hormone replacement 1 year after surgery between PSS and HPA-intact did not reach statistical significance, although stalk sacrifice had a higher risk (OR, 5.633; 95% CI, 0.753–42.133; p = 0.092).

**Table 2:** Univariate logistic regression analysis of need for anterior pituitary hormone replacement 1 year after surgery and permanent diabetes insipidus (DI) based on pre-, intra-, and postoperative data in the training cohort.

Variable	Need for anterior pituitary hormone replacement 1 year after surgery			Permanent DI		
	OR	95% CI	p value	OR	95% CI	p value
Age, y	1.001	0.981–1.022	0.903	0.989	0.970–1.008	0.253
Age group (adult vs. child)	1.179	0.435–3.197	0.747	1.070	0.415–2.762	0.888
Sex (female vs. male)	1.117	0.541–2.310	0.764	1.216	0.618–2.390	0.571
Preop BMI, kg/m^2^	1.006	0.913–1.109	0.903	1.052	0.959–1.153	0.281
Preop hyperprolactinemia (yes vs. no)	0.907	0.468–1.961	0.907	0.707	0.361–1.381	0.310
Preop hydrocephalus (yes vs. no)	1.712	0.672–4.360	0.259	1.557	0.684–3.542	0.291
Preop DI (yes vs. no)	1.813	0.747–4.402	0.188	9.073	3.253–25.305	**<0.001**
Preop pituitary function	–	–	**0.001**	–	–	0.646
Normal	1	–	NA	1	–	NA
Partial hypopituitarism	3.342	1.510–7.397	0.003	1.243	0.589–2.623	0.568
Panhypopituitarism	12.500	2.635–59.295	0.001	1.591	0.589–4.296	0.360
Maximum diameter (≥3 cm vs. < 3 cm)	0.839	0.409–1.717	0.630	1.002	0.514–1.953	0.996
Calcification (yes vs. no)	1.433	0.697–2.946	0.328	1.597	0.815–3.128	0.172
Tumor consistency	–	–	0.171	–	–	0.143
Solid	1	–	NA	1	–	NA
Mixed	1.440	0.591–3.510	0.422	0.823	0.362–1.869	0.641
Cystic	0.630	0.244–1.624	0.339	0.415	0.163–1.056	0.065
Tumor location	–	–	**0.012**	–	–	0.173
Subdiaphragmatic	1	–	NA	1	–	NA
Supradiaphragmatic	0.134	0.030–0.597	0.008	1.620	0.667–3.936	0.287
Pure endoventricular	0.696	0.055–8.748	0.789	5.250	0.900–30.621	0.065
EOR (STR vs. GTR)	0.532	0.153–1.847	0.320	0.512	0.143–1.835	0.304
Postop radiotherapy (yes vs. no)	0.532	0.153–1.847	0.320	0.512	0.143–1.835	0.304
Postop hypernatremia (yes vs. no)	1.862	0.837–4.140	0.127	1.880	0.917–3.854	**0.085**
Pathology (PCP vs. ACP)	0.805	0.223–2.906	0.740	0.770	0.224–2.653	0.679
Injury type	–	–	**<0.001**	–	–	**<0.001**
HPA-intact	1	–	NA	1	–	NA
HI	138.000	17.913–1063.110	<0.001	28.875	6.383–130.630	<0.001
HI+PSS	74.400	13.266–417.254	<0.001	16.500	4.473–60.872	<0.001
HI+PSS+PGI	132.000	10.789–1615.004	<0.001	60.500	6.019–608.126	<0.001
PSS	5.333	0.828–34.337	0.078	1.650	0.310–8.793	0.557
PGI	192.000	16.040–2298.245	<0.001	1.179	0.229–6.076	0.844
PGI+PSS	42.000	4.976–354.537	0.001	1.571	0.235–10.491	0.641

P value with bold font indicates significance. DI, diabetes insipidus; OR, odds ratio; CI, confidence interval; BMI, body mass index; NA, not applicable; EOR, extent of resection; GTR, gross total resection; STR, subtotal resection; ACP, adamantinomatous craniopharyngioma; PCP, papillary craniopharyngioma; HPA, hypothalamus–pituitary axis; HI, hypothalamic injury; HI+PSS, hypothalamic injury combined with pituitary stalk sacrifice; HI+PSS+PGI, hypothalamic injury combined with pituitary stalk sacrifice and pituitary gland injury; PSS, pituitary stalk sacrifice; PGI, pituitary gland injury; PGI+PSS, pituitary gland injury combined with pituitary stalk sacrifice.

**Table 3:** Multivariate logistic regression analysis of need for anterior pituitary hormone replacement 1 year after surgery and permanent diabetes insipidus (DI) in the training cohort.

Variable	Need for anterior pituitary hormone replacement 1 year after surgery			Permanent DI		
	OR	95% CI	p value	OR	95% CI	p value
Preop DI (yes vs. no)	–	–	**–**	8.997	2.580–31.374	**0.001**
Postop hypernatremia (yes vs. no)	–	–	**–**	**–**	**–**	0.414
Preop pituitary function	–	–	**0.021**	**–**	**–**	**–**
Normal	1	–	NA	–	–	—
Partial hypopituitarism	4.184	1.225–14.292	0.022	–	–	–
Panhypopituitarism	13.742	1.345–140.417	0.027	–	–	–
Tumor location	–	–	0.955	–	–	–
Subdiaphragmatic	–	–	–	–	–	–
Supradiaphragmatic	–	–	–	–	–	–
Pure endoventricular	–	–	–	–	–	–
Injury type	–	–	**<0.001**			**<0.001**
HPA-intact	1	–	NA	1	–	
HI	194.174	21.311–1769.253	<0.001	31.393	6.319–155.964	<0.001
HI+PSS	89.443	13.642–586.416	<0.001	12.820	3.138–52.380	<0.001
HI+PSS+PGI	68.111	4.975–932.565	0.002	62.934	5.795–683.512	0.001
PSS	5.633	0.753–42.133	0.092	1.580	0.257–9.707	0.621
PGI	159.790	11.974–2132.357	<0.001	0.980	0.164–5.848	0.982
PGI+PSS	31.921	3.320–306.873	0.003	2.399	0.335–17.192	0.384

P value with bold font indicates significance. DI, diabetes insipidus; OR, odds ratio; CI, confidence interval; NA, not applicable; HPA, hypothalamus–pituitary axis; HI, hypothalamic injury; HI+PSS, hypothalamic injury combined with pituitary stalk sacrifice; HI+PSS+PGI, hypothalamic injury combined with pituitary stalk sacrifice and pituitary gland injury; PSS, pituitary stalk sacrifice; PGI, pituitary gland injury; PGI+PSS, pituitary gland injury combined with pituitary stalk sacrifice.

### Risk Factors for Permanent DI

Similarly, through univariate (**Table 2**) and stepwise multivariate analyses, preop DI (yes vs. no, 8.997 [2.580–31.374]) and the injury type of HPA (for HI vs. HPA-intact, 31.393 [6.319–155.964]; for HI+PSS vs. HPA-intact, 12.820 [3.138–52.380]; for HI+PSS+PGI vs. HPA-intact, 62.934 [5.795–683.512]; for PSS vs. HPA-intact, 1.580 [0.257–9.707]; for PGI vs. HPA-intact, 0.980 [0.164–5.848]; for PSS+PGI vs. HPA-intact, 2.399 [0.335–17.192]) were determined as independent risk factors associated with permanent DI and were used to form a nomogram. Note that when we compared PSS vs. HPA-intact, PGI vs. HPA-intact, and PSS+PGI vs. HPA-intact, the significant difference in the risk of permanent DI was not found (**Table 3**).

### Difference in Endocrinological Deficiency 1 Year After Surgery Between Injury Types

**Figure 2:**
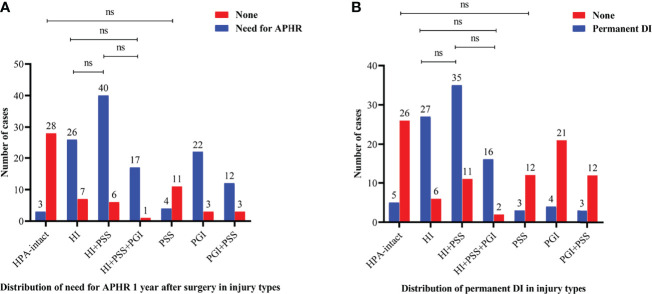
Bar charts showing the distributions of need for anterior pituitary hormone replacement 1 year after surgery **(A)** and permanent DI **(B)** between injury types in the entire cohort. HPA, hypothalamus–pituitary axis; PSS, pituitary stalk sacrifice; PGI+PSS, pituitary gland injury combined with pituitary stalk sacrifice; HI+PSS+PGI, hypothalamic injury combined with pituitary stalk sacrifice and pituitary gland injury; HI+PSS, hypothalamic injury combined with pituitary stalk sacrifice; PGI, pituitary gland injury; HI, hypothalamic injury; APHR, anterior pituitary hormone replacement; DI, diabetes insipidus; ns, not significant.

Further, we investigated the difference in endocrinological deficiency 1 year after surgery between the injury types of the HPA in the entire cohort (**Figure 2**). With regard to anterior pituitary dysfunction, the significant difference in the rate of need for anterior pituitary hormone replacement 1 year after surgery between injury types was found (p < 0.001). In addition, the rate of need for anterior pituitary hormone replacement 1 year after surgery was significantly lower in HPA-intact type and PSS type than HI type, HI+PSS type, HI+PSS+PGI type, PGI type, and PGI+PSS type (p < 0.05), whereas the difference between HPA-intact type and PSS type was not statistically significant (p > 0.05). Intriguingly, the difference in the rate of need for anterior pituitary hormone replacement 1 year after surgery between HI type, HI+PSS type, and HI+PSS+PGI type did not reach statistical significance (p > 0.05, **Figure 2A**). Regarding posterior pituitary dysfunction, there was a significant difference in the rate of permanent DI between injury types (p < 0.001). Moreover, the rate of permanent DI was significantly lower in HPA-intact type, PSS type, PGI type, and PGI+PSS type than HI type, HI+PSS type, and HI+PSS+PGI type (p < 0.05), whereas the difference between HPA-intact type, PSS type, PGI type, and PGI+PSS type was not statistically significant (p > 0.05). Likewise, the difference in the rate of permanent DI between HI type, HI+PSS type, and HI+PSS+PGI type did not reach statistical significance (p > 0.05, **Figure 2B**).

### Nomograms and Model Performance

**Figure 3:**
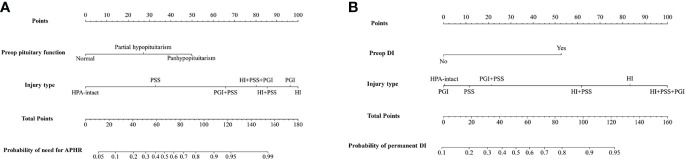
Nomograms to predict the rate of need for anterior pituitary hormone replacement 1 year after surgery **(A)** and permanent diabetes insipidus **(B)** in patients with craniopharyngioma. HPA, hypothalamus–pituitary axis; PSS, pituitary stalk sacrifice; PGI+PSS, pituitary gland injury combined with pituitary stalk sacrifice; HI+PSS+PGI, hypothalamic injury combined with pituitary stalk sacrifice and pituitary gland injury; HI+PSS, hypothalamic injury combined with pituitary stalk sacrifice; PGI, pituitary gland injury; HI, hypothalamic injury; APHR, anterior pituitary hormone replacement; DI, diabetes insipidus.

Nomograms based on the aforementioned independent risk factors to predict the probability of need for anterior pituitary hormone replacement 1 year after surgery and permanent DI are shown in **Figure 3**. The predicted probability of need for anterior pituitary hormone replacement 1 year after surgery and permanent DI for CP patients could be obtained, based on the sum of each variable score. Higher total scores calculated from the nomograms were associated with higher risk to suffer from endocrinological deficiency 1 year after surgery. For example, a CP patient with preoperative partial hypopituitarism, DI, and intraoperative pituitary stalk sacrifice would have a total of 110 scores (50 scores for partial hypopituitarism and 60 scores for pituitary stalk sacrifice) and a total of 92 scores (84 scores for preoperative DI and 18 scores for pituitary sacrifice), for a predicted need for anterior pituitary hormone replacement 1 year after surgery and permanent DI of 90.0% and 84.0%, respectively.

**Figure 4:**
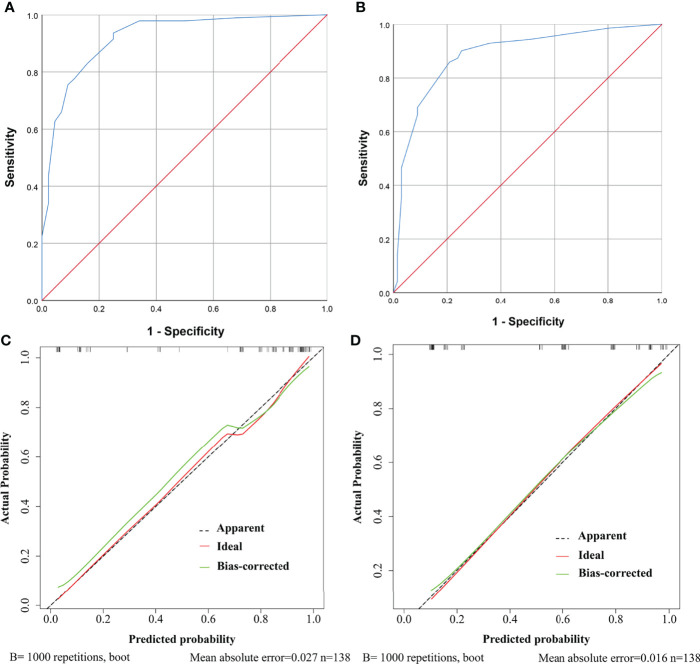
ROC curves and calibration curves of the models showing predictive performance for need for anterior pituitary hormone replacement 1 year after surgery and permanent diabetes insipidus (DI) in the training cohort. **(A)** The AUC of the model for need for anterior pituitary hormone replacement is 0.921, with 95% CI ranging from 0.871 to 0.970. **(B)** The AUC of the model for permanent DI is 0.885, with 95% CI ranging from 0.827 to 0.943. **(C)** Calibration curve of the prediction model for need for anterior pituitary hormone replacement. The grey dashed line represents the apparent curve (non-correction), the green line represents the bias-correction curve, and the red line represents the ideal curve. B = 1,000 repetitions; n = 138, mean absolute error = 0.027. **(D)** As in **(C)** but for permanent DI.

**Figure 5:**
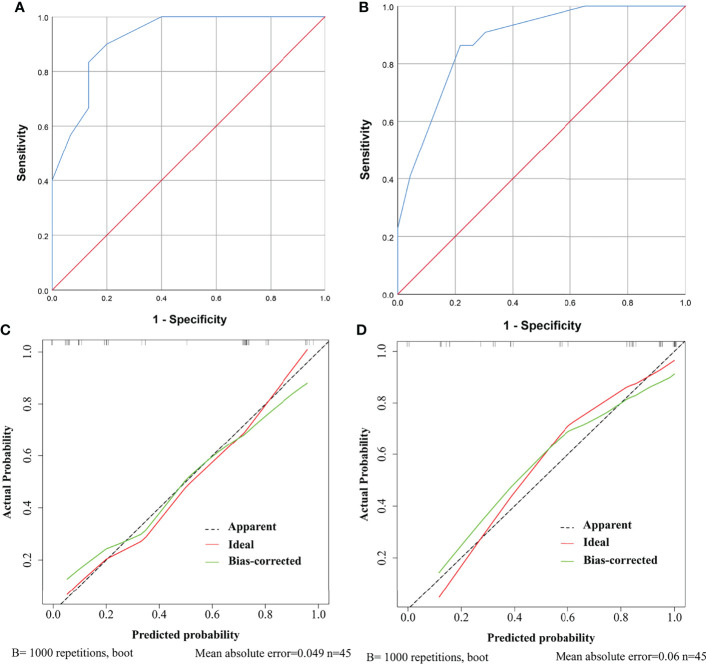
ROC curves and calibration curves of the models showing predictive performance for need for anterior pituitary hormone replacement 1 year after surgery and permanent diabetes insipidus (DI) in the validation cohort. **(A)** The AUC of the model for need for anterior pituitary hormone replacement is 0.921, with 95% CI ranging from 0.834 to 1.000. **(B)** The AUC of the model for permanent DI is 0.880, with 95% CI ranging from 0.782 to 0.979. **(C)** Calibration curve of the prediction model for need for anterior pituitary hormone replacement. The grey dashed line represents the apparent curve (non-correction), the green line represents the bias-correction curve, and the red line represents the ideal curve. B = 1,000 repetitions; n = 45, mean absolute error = 0.049. **(D)** As in **(C)** but for permanent DI.

Further, ROC curves with AUCs and calibration curves were used to evaluate the performance of the nomograms. In the training cohort, for need for anterior pituitary hormone replacement 1 year after surgery and permanent DI predictions, the nomograms showed good discriminative abilities with AUCs of 0.921 and 0.885, respectively (**Figures 4A, B**). Calibration curves showed that the prediction curves were appropriately consistent with the ideal ones, which indicated that the nomograms had good calibration powers (**Figures 4C, D**). In addition, we validated the stability of the nomograms in the validation cohort. Similarly, good discriminative abilities with AUCs of 0.921 and 0.880 and moderate calibration powers of the nomograms were indicated by the ROC curves (**Figures 5A, B**) and calibration plots (**Figures 5C, D**), respectively.

## Discussion

*Via* categorizing the injury of the HPA into seven types, our study shows that although the stalk and the pituitary were anatomically preserved, relative to the HPA-intact type, the exclusive injury of the hypothalamus could significantly increase the risk of endocrinological deficiency 1 year after surgery. In addition, when the hypothalamus and the pituitary were intact, relative to the HPA-intact type, sacrificing the stalk infiltrated by tumors did not significantly increase the risk of anterior pituitary dysfunction 1 year after CP surgery. Meanwhile, when the hypothalamus was intact, relative to HPA intact, sacrificing the PS infiltrated by CPs did not significantly increase the risk of permanent DI. By contrast, when the hypothalamus was injured, sacrificing the stalk infiltrated by CPs did not have a significantly worse pituitary function 1 year after surgery than stalk preservation. Based on aforementioned results, we conclude that the role of the hypothalamus in maintaining pituitary function is critical and the stalk infiltrated by CPs could be sacrificed to achieve GTR, not substantially resulting in significantly worse endocrinological efficiency 1 year after surgery. Additionally, *via* multivariate analysis, we found that preop pituitary function and the injury type of the HPA were independent risk factors for need for anterior pituitary hormone replacement 1 year after surgery, while preop DI and the injury type of the HPA were independent risk factors for permanent DI. At last, for the first time, we developed nomograms harboring good discriminations and calibration powers to predict endocrinological deficiency 1 year after CP resection.

As published clinical literature evaluating the ramifications of the hypothalamus damage after CP surgery mainly focused on hypothalamic obesity, cognitive dysfunction, sleep disorders, and impaired temperature regulation, knowledge about the effects of exclusive hypothalamus injury on pituitary function is limited (12, 24, 25). *Via* focusing on the effects of exclusive injury of the hypothalamus on anterior pituitary function after surgery, we found that patients suffering from exclusive hypothalamus injury had a significantly increased rate of need for anterior pituitary hormone replacement 1 year after surgery, which shows a critical role of the intact hypothalamus in maintaining anterior pituitary function. Moreover, we also found that exclusively sacrificing the stalk infiltrated by tumors would not significantly increase the rate of need for anterior pituitary hormone replacement 1 year after surgery. Not completely consistent with our results, previous studies reported that sacrificing the stalk infiltrated by tumors could result in worse anterior pituitary function after CP surgery (7, 10, 11). The following reasons may account for this discrepancy. First, the results in our study are based on the premise that the hypothalamus and the pituitary are intact, which is not mentioned in aforementioned studies. Second, some previous studies do not report whether or not the difference in anterior pituitary function after surgery between stalk sacrifice and stalk preservation has reached statistical significance (10, 11). At last, the different duration of follow-up is another reason. The following underlying mechanisms are thought to account for our results. First, hypothalamic releasing factors (HRFs) produced in the hypothalamus are transported along neurons to the median eminence, where they are secreted into the portal vein and sequentially stimulate the anterior pituitary to synthesize and release anterior pituitary hormones (26–30). Second, previous studies have reported that when the stalk is resected, portal vein recanalization could occur over a long period of time demonstrated by MR images (31, 32). Thus, based on aforementioned evidence, we speculate that the maintaining of anterior pituitary function 1 year after stalk sacrifice is attributed to the fact that through the preserved median eminence, the HRFs are secreted into the reestablished portal vein and sequentially act on the preserved anterior pituitary lobe (32). At last, it must be highlighted that according to our surgical strategies, the stalk sacrificed has been infiltrated or destroyed by CPs before surgery, so some degree of compensation may have existed. Nevertheless, our results require more multicenter clinical studies with a large sample size to validate.

Likewise, regarding posterior pituitary dysfunction after CP surgery, we found that the role of the intact hypothalamus in maintaining posterior pituitary function is critical and resecting the stalk below the level of the median eminence could not significantly increase the rate of permanent DI. In accordance with our results, many studies have reported that low-level stalk transection usually could not result in permanent DI (18, 32–36). The maintaining of posterior pituitary function after sacrificing stalk infiltrated by tumors may attribute to being hypersensitive to plasma-intrinsic antidiuretic hormone (ADH), occurrence of ectopic posterior lobe, and portal vein recanalization (31, 32, 35, 37). As to the underlying compensatory mechanism for DI, based on a rat experiment, Feng et al. (38) speculated that the functional ectopic posterior lobe increased the GAP-43 expression *via* PI3K/AKT pathways to alleviate DI after stalk sacrifice. Likewise, more clinical and laboratory studies are needed to elucidate the accurate compensatory mechanism for DI after stalk sacrifice.

In accordance with our results, preoperative pituitary dysfunction as a risk factor associated with endocrinological deficiency during follow-up has been widely reported, as preexisting endocrinological deficiency of CP patients undergoing surgery is usually only mildly improved during follow-up (9, 39, 40). There is an agreement in the literature that radiotherapy can worsen pituitary function (5, 7, 41, 42). Intriguingly, our statistical analysis excluded the role of radiotherapy as an independent risk factor associated with endocrinological deficiency 1 year after surgery. Limited sample size and relatively short time interval from receiving radiotherapy to the endpoint of our study may account for our results. In addition, some studies (43, 44) have reported that the EOR could affect endocrinological outcomes, which is not confirmed by our study. One possible explanation is that in the context that the goal of our surgery is to resect as much tumors as possible, the factors determining GTR or STR, such as adherence of CP to critical vessels, do not lead to a difference in the manipulation of HPA between GTR and STR. Thus, EOR (STR vs. GTR) has no significant difference in endocrinological outcomes, which is in accordance with previous studies (7, 10).

Identifying independent risk factors is of great importance to comprehensively understand the prognosis of a given disease. Further, a nomogram based on independent risk factors can provide more individual and accurate prognosis data for treating clinicians and patients or their relatives. In the last two decades, neurosurgical clinical prediction models have increased exponentially, for a variety of clinical outcomes (23, 45). This is the first study to construct models to predict postoperative endocrinological prognosis in patients with CP. Our nomograms were constructed following standard procedures and harbored good discriminations and calibration powers, which merits application in clinical works.

### Limitations

First, due to the retrospective nature of the study, selection bias inevitably exists. Second, the present findings were based on limited data from a single center and on patients only undergoing EEA, it is necessary to validate them in multiple centers with a large sample size. Particularly, attention must be paid when interpreting our results that pituitary stalk infiltrated by tumors could be sacrificed, as only 15 patients underwent exclusive stalk sacrifice, warranting further investigation. Third, we defined endocrinological deficiency as the need for pituitary hormone replacement rather than objective measurement of pituitary hormone levels, which may underestimate the rate of pituitary deficiency, although it arguably gives a more clinically meaningful result (14). At last, the injury type of the HPA cannot be quantitatively defined, which may lead to bias when determining injury types between different neurosurgeons.

## Conclusion

Collectively, our preliminary study suggests that the role of the intact hypothalamus in maintaining pituitary function is critical and the pituitary stalk infiltrated by CPs could be sacrificed to achieve GTR, not substantially resulting in significantly worse endocrinological efficiency 1 year after surgery. Moreover, our study highlights that more clinical and laboratory studies are required to elucidate the accurate mechanism and validate our results. The established nomograms with good predictive performance can provide a user-friendly tool to predict the rate of hypopituitarism 1 year after surgery, which is helpful for clinicians to better counsel patients and provide anticipatory guidance on postoperative expectations and management.

## Data Availability Statement

The original contributions presented in the study are included in the article. Further inquiries can be directed to the corresponding author.

## Ethics Statement

The studies involving human participants were reviewed and approved by the Institutional Ethics Committee of the First Affiliated Hospital of Nanchang University. For this retrospective study, formal consent was not required.

## Author Contributions

TH and JW contributed to the study’s conception and design. Analysis of the data was performed by TH, JW, XW, and LY. Material preparation and data collection were performed by JW, XW, LY, SHX, BT, ZGT, BWW, HD, YYB, LZ, and YQY. The first draft of the manuscript was written by JW, and all authors commented on previous versions of the manuscript. All authors contributed to the article and approved the submitted version.

## Funding

This work was supported by the National Natural Science Foundation of China (grant nos. 82060246 and 81460381), Ganpo555 engineering excellence of the Jiangxi Science and Technology Department (2013), and the Key Research and Invention Plan of Jiangxi Science and Technology Department (20192BBG70026).

## Conflict of Interest

The authors declare that the research was conducted in the absence of any commercial or financial relationships that could be construed as a potential conflict of interest.

## Publisher’s Note

All claims expressed in this article are solely those of the authors and do not necessarily represent those of their affiliated organizations, or those of the publisher, the editors and the reviewers. Any product that may be evaluated in this article, or claim that may be made by its manufacturer, is not guaranteed or endorsed by the publisher.
